# COVID-19-Associated Cognitive Biases on Pneumonia Differential Diagnosis

**DOI:** 10.7759/cureus.55144

**Published:** 2024-02-28

**Authors:** Pedro Cruz, Ana M Meireles, Marina Santos, Maria R Rodrigues

**Affiliations:** 1 Medical Oncology, Portuguese Oncology Institute of Porto, Porto, PRT; 2 Hematological Oncology, Portuguese Oncology Institute of Porto, Porto, PRT; 3 Internal Medicine, Portuguese Oncology Institute of Porto, Porto, PRT

**Keywords:** organizing pneumonia, clinical case report, pneumonia, radiation-induced organizing pneumonia, heuristics and biases, covid-19

## Abstract

The coronavirus disease 2019 (COVID-19) pandemic favors cognitive biases such as anchoring and availability biases. The first refers to overvaluing some of the initial information and establishing a diagnosis too early, with resistance to future adjustments. The latter happens when diagnoses more frequently considered are regarded as more common in reality. This case, in which the correct diagnosis was delayed due to these biases, highlights the need to remain aware of them as a means toward timely diagnosis and therapeutic success of pneumonia cases.

An 84-year-old woman presented with a mild non-productive cough for two months and fever. She had a history of breast carcinoma treated with radiotherapy in the previous year. Computerized tomography (CT) showed extensive bilateral consolidation foci with ground-glass-opacification areas and bilateral pleural effusion, CO-RADS 3.

COVID-19 with bacterial superinfection was suspected and levofloxacin was initiated. Nasopharyngeal swab polymerase chain reaction (PCR) was carried out three times, always negative for SARS-CoV-2. As the patient remained with fever and cough, the antibiotic was escalated to piperacillin/tazobactam and then to meropenem/vancomycin. She underwent bronchofibroscopy and alveolar lavage, with negative SARS-CoV-2 PCR. The re-evaluation CT scan maintained bilateral consolidations, with an aerial bronchogram. The biopsy of pulmonary consolidation allowed the diagnosis of radiation-induced organizing pneumonia. Prednisolone was initiated and achieved clinical remission and radiological improvement.

This case highlights the need to remain aware of cognitive biases both when COVID-19 is suspected or ruled out and to consider other diagnoses when there is a lack of therapeutic response.

## Introduction

The coronavirus disease 2019 (COVID-19) pandemic is causing a great impact on medical services worldwide. The threat of this new, devastating disease favors cognitive biases which can be detrimental [1]. Two particularly important are anchoring and availability biases. The first refers to overvaluing some of the initial information or even establishing a diagnostic hypothesis too early, creating resistance to future adjustments that new information might warrant. The latter refers to considering diagnoses more frequently thought of as more frequent in reality [2]. This case highlights the need to remain aware of these potential biases as a means toward timely diagnosis and therapeutic success of pneumonia cases.

## Case presentation

An 84-year-old woman, with a history of breast carcinoma treated in the previous year, underwent a surveillance computerized tomography (CT) which was suggestive of COVID-19. She was admitted for further investigation of severe bilateral pneumonia.

She had a mild non-productive cough, mostly in the morning, with progressive astheny and anorexia for two months, which had been investigated by her general practitioner who identified normocytic normochromic anemia one month ago. She denied dyspnea, rhinorrhea, odynophagia, nausea, vomiting, pain, gastrointestinal or genitourinary complaints, or contact with suspected COVID-19 patients.

Her medical history included invasive breast carcinoma of no special type in the previous year treated with partial mastectomy and adjuvant radiotherapy, having initiated adjuvant hormonal therapy. She had no smoking history (active or passive). Her regular medications were iron (III)-hydroxide polymaltose complex, perindopril, lansoprazole, alprazolam, calcium carbonate, cholecalciferol, and anastrozole.

She was a widow, living alone in her house in an urban area, with frequent support from her sons, and was a retired household servant. She had a family history of cancer in four siblings (central nervous system, colorectal, oral cavity, and soft tissue sarcoma).

On examination, she was febrile (38 ºC), hemodynamically stable, and eupneic with no respiratory distress signs and peripheral oxygen saturation 96% on ambient air. Breath sounds were diminished in the left pulmonary base, with rhonchi and crackles in both pulmonary bases and a normal inspiratory/expiratory ratio.

**Figure 1:**
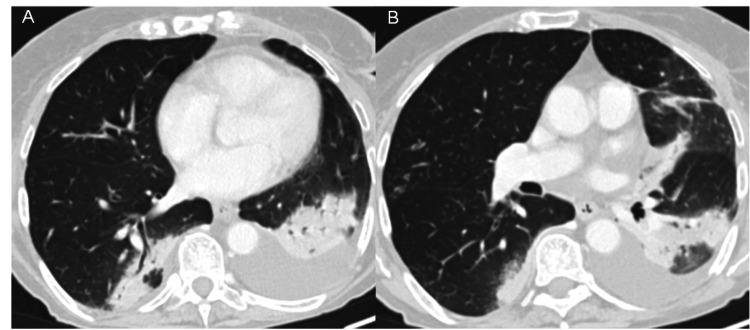
Chest CT at admission. Extensive bilateral consolidation foci, ground-glass-opacification areas in the left superior lobe, left inferior lobe and right inferior lobe, and pleural effusion.

The arterial blood gas analysis on ambient air revealed pH 7.44, partial pressure of carbon dioxide 35 mmHg, partial pressure of oxygen 68 mmHg, and HCO_3_^-^ 23.8 mmol/L. The contrast CT (Figure 1) showed extensive bilateral consolidation foci with ground-glass-opacification areas in the left superior lobe, left inferior lobe, and right inferior lobe, tree-in-bud micronodules in the right lung, peripheral ground-glass-opacification foci in the right superior lobe, and bilateral pleural effusion (larger on the left side), suggesting an inflammatory or infectious etiology. CO-RADS 3: unsure [3].

The nasopharyngeal swab polymerase chain reaction (PCR) was negative for SARS-CoV-2 at presentation. Bacterial pneumonia was assumed, blood and sputum cultures were collected, levofloxacin was initiated and the patient was admitted.

A nasopharyngeal swab PCR for SARS-CoV-2 24 hours after admission was again negative. Test results for *Influenza *and Respiratory Syncytial viruses, *Legionella, *and Pneumococcal urinary antigens were negative.

After four days of antibiotics, the patient remained with fever and cough, thus raising the suspicion of a false negative SARS-CoV-2 PCR. She underwent a third nasopharyngeal swab PCR, again negative, and the antibiotic was escalated to piperacillin/tazobactam 4.5 g *qid* for six days. No microbiological agent was isolated on sputum nor blood, PCR for *M. tuberculosis*, *P. jirovecii,* and *Aspergillus* in sputum, as well as Ziehl-Neelsen staining, was negative.

On day 7 after admission, she underwent bronchofibroscopy and alveolar lavage: SARS-CoV-2 PCR was negative, no microbiological or mycobacterial agent was isolated on culture, *Legionella* and *Aspergillus* antigen were negative, while its cytology was compatible with an inflammatory/infectious process with no evidence of malignancy.

**Figure 2:**
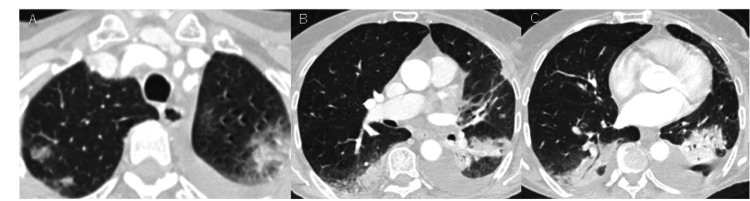
Chest CT on day 8 after admission. Extensive pulmonary condensations.

**Figure 3:**
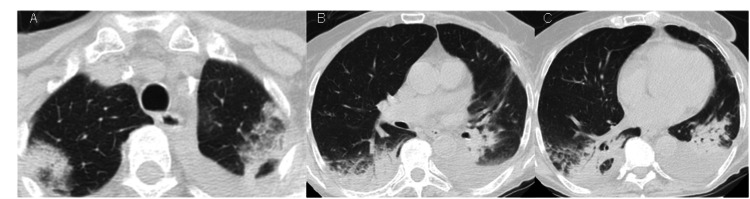
Chest CT on day 14 after admission. Persisting bilateral pulmonary consolidations with aerial bronchogram despite antibiotic therapy escalation.

On day 8 after admission, she remained with fever and cough and a re-evaluation CT scan (Figure 2) showed persisting extensive foci of pulmonary condensations. On day 10, she escalated to meropenem/vancomycin for 11 days. On day 14, another re-evaluation CT scan (Figure 3) maintained bilateral consolidations, with an aerial bronchogram.

On day 20 after admission, biopsy of a pulmonary consolidation was performed, revealing organizing pneumonia, and the diagnosis of radiation-induced organizing pneumonia (RIOP) was established.

The patient began prednisolone 40 mg *qd* and was discharged. Fifty-one days afterward, she had clinical remission and radiological improvement and was initiated steroid tapering.

**Figure 4:**
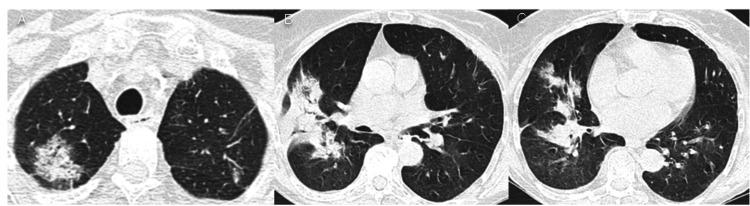
Chest CT five months after hospital discharge. Migratory pulmonary consolidations.

**Figure 5:**
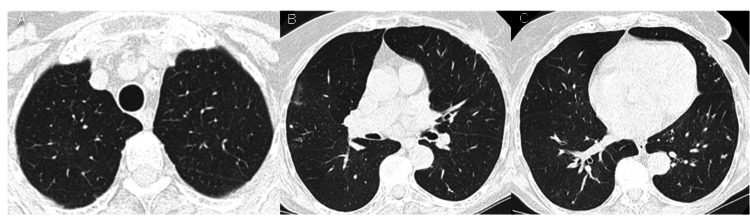
Chest CT 11 months after hospital discharge. Regressing pulmonary consolidations with corticotherapy.

During follow-up, the patient remained asymptomatic. Chest CT revealed pulmonary migratory consolidations with decreasing overall size, as expected in RIOP, at 5 (Figure 4) and 11 (Figure 5) months after hospital discharge.

**Figure 6:**
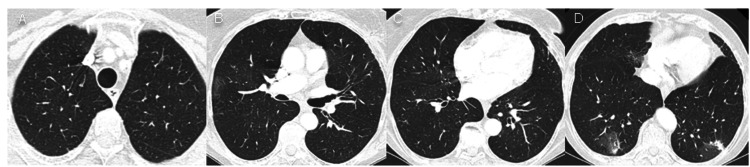
Chest CT 18 months after hospital discharge. Relapsing pulmonary consolidations and ground-glass-opacification areas in the lower lobes after steroid tapering.

**Figure 7:**
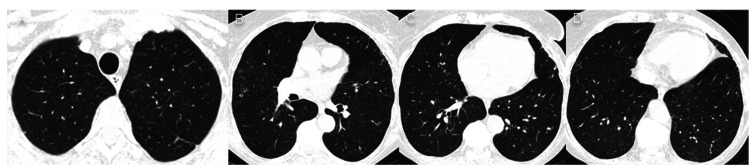
Chest CT 21 months after hospital discharge. Resolution of pulmonary condensations and ground-glass-opacification areas with corticotherapy.

At 18 months however, the CT (Figure 6) showed an increase in the overall size of pulmonary condensations with ground-glass, which is frequent when corticosteroids are stopped [4], and so she reinitiated prednisolone 40 mg *qd*, with good clinical and radiological response at 21 months (Figure 7).

## Discussion

This case is a good example of cognitive biases in daily medical practice, which have been associated with the COVID-19 pandemic [1], namely anchoring and availability biases.

Anchoring bias, that is the assumption of a diagnosis early and resisting adjustments in light of new information [2], was very clear in this case. Fever, mild non-productive cough, respiratory insufficiency, and pulmonary consolidations led to the assumption of COVID-19 with superinfection with bacterial pneumonia. This assumption remained from admission until eight days afterward, even after the first negative PCR tests and ineffective antibiotics, although the history was very typical of RIOP. In fact, the patient fulfilled all of the classic diagnostic criteria except for the no longer applicable lack of specific cause: breast radiotherapy within 12 months, respiratory and/or general symptoms for at least two weeks, and lung infiltrates in non-irradiated areas [5].

RIOP, a type of secondary organizing pneumonia, as opposed to cryptogenic organizing pneumonia (COP, previously called bronchiolitis obliterans organizing pneumonia (BOOP)) [5], occurs mainly in breast cancer patients. It causes migratory lung infiltrates (consolidation possibly with air-bronchogram, ground-glass, and/or nodular opacities) and is frequently confused with infectious pneumonia [5-8]. Its incidence has been described as between 1.7% and 3% after radiotherapy including the breast [5,9]. Relapses, possibly multiple, occur in 13 to 58% of cases, but they do not increase mortality or functional morbidity [4,8,10].

Availability bias occurs when diagnoses more frequently considered are deemed as occurring more commonly. The more thought-of COVID-19 and bacterial pneumonia dominated the mindset of the attending physicians initially, while the rare RIOP was not regarded until late in the antibiotic escalation. The CT alterations also contributed to the confounding setting, as they could be associated with a worse prognosis for COVID-19 thus compelling the physicians to escalate the antibiotic more promptly. Furthermore, it is important to remember that a CO-RADS 3 classification means that the CT alterations are equivocal as they are associated with other causes of pneumonia, including non-infectious ones such as RIOP [3].

One limitation of this case is the lack of cellular counts in the bronchoalveolar lavage fluid, namely of eosinophils, lymphocytes, and mast cells to better characterize RIOP and exclude chronic eosinophilic pneumonia, a very similar disease with eosinophilia and increased eosinophil count in the bronchoalveolar fluid also treated with steroids. However, it occurs in patients with asthma or atopy [5,11] and is thus unlikely in this case.

## Conclusions

This case highlights the need to remain aware of cognitive biases both when COVID-19 is suspected and when it is ruled out and to consider other diagnostic hypotheses in face of an unexpected lack of therapeutic response.
